# Evaluation of sOX-2 levels in type2 diabetic foot and nephropathic patients: association with disease activity

**DOI:** 10.1186/1939-4551-8-S1-A9

**Published:** 2015-04-08

**Authors:** Arzu Didem Yalcin, Hasan Onur Arik, Betul Celik, Derya Seyman, Gulsum Tetik, Bensu Gursoy, Sukran Kose, Saadet Gumuslu

**Affiliations:** 1 Internal Medicine, Allergy and Clinical Immunology, Genomics Research Center, Academia Sinica,11529, Taipei, Taiwan; 2 Orthopaedics and Traumatology Clinic, Yozgat State Hospital, Turkey; 3 Department of Laboratory Medicine and Pathology, Mayo Clinic in Jacksonville, USA; 4 Department of Infectious Diseases and Clinical Microbiology, Antalya Education and Research Hospital, Turkey; 5 Department of Plastic Surgery, Antalya Education and Research Hospital, Turkey; 6 Infection Disease Clinic, Tekirdag State Hospital, Turkey; 7 Department of Infectious Diseases and Clinical Microbiology, Allergy and Clinical Immunology Unit, Tepecik Education and Research Hospital, Izmir, Turkey; 8 Department of Medical Biochemistry, Faculty of Medicine, Akdeniz University, 07070, Antalya, Turkey

## Background

OX-2 is a member of the immunoglobulin supergene family of receptors . sOX-2 was originally described as a myeloid receptor, being expressed on macrophages, granulocytes and dendritic cells, and also expressed on T cells, Bcells, and natural killer cells. It displays a restricted tissue distribution, including activated T and B cells. sOX-2 is induced by inflammatory cytokines, including TNF- and bindsto OX-2 receptor.We evaluated other biomarkers like high-sensitivity CRP(hs-CRP) in DFI patients to compare with that of healthy controls. Possible correlationswere investigated between these markers and creatinine levels, Wagner grading system(WGS), and body mass index(BMI), as well as sedimentation rate, preprandial glucose levels, and age.

## Methods

We enrolled 23 healthy controls (group A) and 22 T2DM-DFI patients (group B). Group B patients had diabetic nephropathy and foot disease. The T2DM-DFI definition was infection, ulceration, or destruction of deep tissues of the foot associated with neuropathy and/or peripheral arterial disease in the lower extremity of people with diabetes.

## Results

The demographics of the analyzed patients are shown in Table 1. Group B had the following values: DM period: 27.9±10.3 year [mean ±SD], HbA1c: 9.52±0.44% (normal range: 4–6%) and WGS: 1.61 (5 patients with grade 1, 5 patients with grade 2, 7 patients with grade 3, and 5 patient with grade 4). The sOX-2 level in the patient group was 173.8±3.1[mean ±SEM] and in the healthy control group it was 70.52±1.2 [mean ±SEM]. [p<0.0001) (Figure 1). The HbA1c, BUN, creatinine, hs-CRP levels, and sedimentation rates were higher in the patient group (p<0.0001, p<0.001, p<0.001, p<0.005, and p<0.0001, respectively). There was a positive correlation between sOX-2 levels and BUN and creatinine rate values (p<0.05), (r1=0.498; r2=0.675). There was a positive correlation between: HbA1c values and CRP, preprandial glucose, postprandial glucose, and sedimentation rate values (p<0.01, respectively r1 0.479; r2: 0.549; r3: 0.486, r4: 0.858); and between hs-CRP values and BMI values (p<0.05; r: 0.622). In subgroup analysis of T2DM-DFI patients we noticed that sOX-2 levels were higher in WGS I and II patients than in WGS III and IV patients.

## Conclusions

All these results show that the levels of sOX-2 were higher in macrovascular complication of DM as DFI than in autoimmune diaseases and inflamatory skin disorders. Thus, we suggest that there were vascular, immunologic, and neurologic components in DFI whereas autoimmune diaseases and inflamatory skin disorders had only an immunologic component. This should be the evidence of has sOX-2 major pro-inflammatory effect in vascular complication.

